# Forest conservation effectiveness of community forests may decline in the future: Evidence from Cambodia

**DOI:** 10.1093/pnasnexus/pgad320

**Published:** 2023-09-28

**Authors:** Miwa Ota, Tetsuji Ota, Katsuto Shimizu, Nariaki Onda, Vuthy Ma, Heng Sokh, Nobuya Mizoue

**Affiliations:** Graduate School of Bioresource and Bioenvironmental Sciences, Kyushu University, Fukuoka, Fukuoka, 819-0395, Japan; Faculty of Agriculture, Kyushu University, Fukuoka, Fukuoka, 819-0395, Japan; Department of Forest Management, Forestry and Forest Products Research Institute, Tsukuba, Ibaraki, 305-8687, Japan; Tohoku Research Center, Forestry and Forest Products Research Institute, Morioka, Iwate, 020-0123, Japan; Forest-Wildlife Research and Development Institute, Forestry Administration, Khan Sen Sok, Phnom Penh, 120806, Cambodia; Forest-Wildlife Research and Development Institute, Forestry Administration, Khan Sen Sok, Phnom Penh, 120806, Cambodia; Faculty of Agriculture, Kyushu University, Fukuoka, Fukuoka, 819-0395, Japan

**Keywords:** deforestation, forest degradation, community forest, interrupted time series

## Abstract

Community forests (CFs) have been widely established in tropical countries as a tool to achieve forest conservation. Many studies have shown that CFs can contribute to the reduction of deforestation, yet studies that evaluate the contribution of CFs to reducing forest degradation and facilitating forest recovery remain scarce. We investigated the ability of CFs to prevent deforestation and forest degradation and to facilitate forest recovery by using a country-scale longitudinal tree canopy cover and forest cover data set in Cambodia. We found that CFs can prevent both forest degradation and deforestation, but we did not observe a forest recovery effect. We also found that recently established CFs are not effective for forest conservation compared with older CFs. We conclude that, to date, CFs are an effective forest conservation tool; however, this does not necessarily mean that new CFs will be as effective as established ones.

**Significance Statement**

Community forests (CFs) are forest management for achieving forest conservation, and their number is increasing in tropical regions. However, contributions of CFs to preventing forest degradation and facilitating forest recovery have not been quantified. We test the effectiveness of CF on forest conservation in terms of deforestation and forest degradation by using a country-scale longitudinal data set in Cambodia. Deforestation and forest degradation were reduced after CF establishment in the CFs compared with the control sample outside the CFs on average, but the conservation effect declined in the recent CFs. CF currently has a conservation effectiveness, but if the number of CFs with low conservation effect keeps increasing, the conservation effect of CFs may disappear.

## Introduction

Forests perform immensely important ecosystem services because they have high biodiversity values, contribute to local livelihoods, and play a key role in the carbon cycle and climate (1–6). Deforestation and forest degradation hinder these ecosystem services (7–11); thus, preventing deforestation and forest degradation and restoring deforested lands are globally prioritized by international agendas such as the Sustainable Development Goals and Bonn Challenge. Tropical forests occupy a considerably large proportion of the remaining forest area globally but have faced severe deforestation and forest degradation over the past few decades (12). Thus, appropriate forest management that prevents deforestation and forest degradation and facilitates recovery of deforested land is a major challenge for countries that have tropical forests (13).

Community forests (CFs), in which local community members are involved in forest management, are widely established in tropical countries as a tool to achieve forest conservation, as well as livelihood improvement for local people. While some studies have shown a weak contribution of CFs in preventing deforestation (14), a substantial number of studies have shown that CFs can contribute to biodiversity conservation (15), carbon storage (16), and forest conservation (17–20). Yet, a majority of studies only focused on the reduction of deforestation, and contributions of CFs to preventing forest degradation have not been quantified. The area of forest degradation is greater than that of deforestation in the tropics (21, 22), and forest degradation accounts for a substantial proportion of carbon emissions from tropical forests (8). Thus, evaluating the forest conservation effectiveness in terms of both forest degradation and deforestation is more appropriate than evaluating forest conservation with a focus only on deforestation. Contributions of CFs to facilitating forest recovery have also not been quantified. Some analyses considered the contribution of CFs to forest recovery, but these studies qualitatively evaluated the forest conditions of CFs based on three ordinal levels, which were decrease, no change, and increase, without consideration of the time since CFs were established (23–25).

Evaluating forest degradation and forest recovery is more challenging than evaluating deforestation (26). This is because forest degradation and forest recovery are usually gradual processes (26–29), while deforestation is a distinct change from forest to nonforest. Thus, longitudinal analysis that captures gradual and successional forest cover change is crucial to analyze the forest degradation and forest recovery (30). Here, we use longitudinal analysis to assess forest change before and after CF establishment by utilizing a data set that can be used to quantify deforestation, degradation, and recovery via data derived from satellite remote sensing at a country scale.

We aimed to investigate the effect of preventing deforestation and forest degradation and facilitating forest recovery of CFs by using a country-scale longitudinal tree canopy cover (TCC) and forest cover data set from Cambodia, which is experiencing rapid deforestation and forest degradation and plans to establish 2 million ha of CFs by 2029 (31). We applied a controlled longitudinal study design using an annual TCC and forest cover data set at a 30-m resolution from 1989 to 2019 as well as potentially confounding variables and the data for CFs established between 1994 and 2010. We randomly selected the sample locations and conducted statistical matching analysis that controls for potential geographical drivers affecting both outcomes (i.e. TCC and forest cover) and spatial distribution of CFs, with respect to each CF establishment year. Then, we generated trajectories of differences in TCC and forest cover between CFs and non-CFs before and after CF establishment from the matched samples. Finally, we assessed the reduction in forest degradation and deforestation and forest recovery effectiveness of CFs by evaluating the changes of trajectories before and after the establishment of CFs using the interrupted time series (ITS) approach. The ITS approach, which is a kind of segment regression analysis, assesses the effect of intervention (i.e. CF establishment) after controlling for the preexisting trend in a time series outcome from the data collected at regular intervals before and after intervention (32). In addition, we evaluated the effect of CFs on deforestation, forest degradation, and forest recovery after CF establishment using regression analyses with respect to each initial land condition when the CFs were established. We also assessed the effect of the CF establishment year on deforestation, forest degradation, and forest recovery. Our analysis provides a comprehensive insight about forest conservation effect of CFs, including reducing deforestation and forest degradation and facilitating forest recovery, with respect to each CF establishment year.

## Results

In this study, we used two outcomes, TCC and forest cover, to assess the effectiveness of CFs. TCC is the tree canopy cover (0–100%) in a pixel, and forest cover is the binary value that represents forest and nonforest. Forest degradation was measured using TCC as an indicator (a commonly used measure of this process, e.g. 30), and forest cover was used as the indicator of deforestation. In cases of forest recovery, we used both TCC and forest cover.

**Fig. 1.**
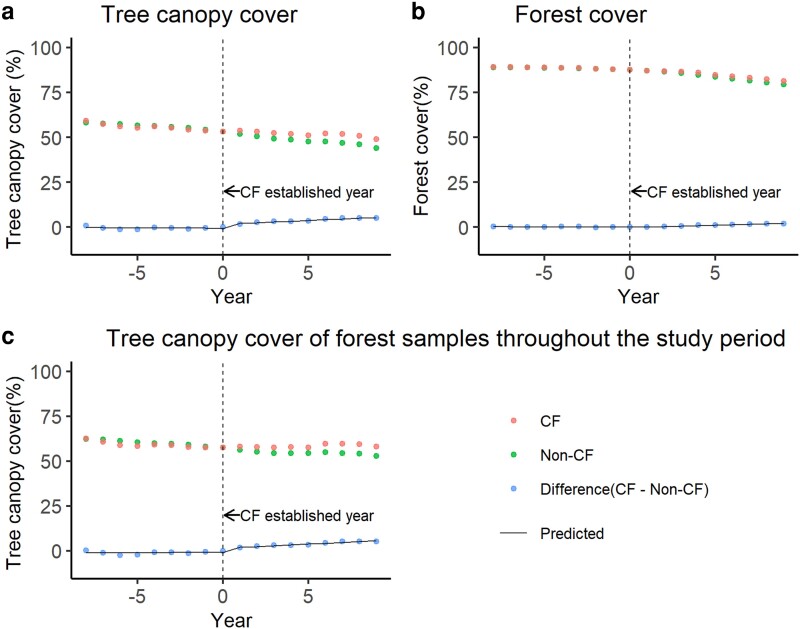
Temporal trends of TCC and forest cover in CFs and non-CFs for matched samples. a) TCC, b) forest cover, and c) TCC derived only from forest samples throughout the study period.

After the statistical matching process, we used the matched samples where the balance of potentially confounding variables was acceptable. We used an absolute standardized mean difference (SMD) to assess the balance of potentially confounding variables and considered an absolute SMD of less than 0.25 after matching as acceptable. Finally, we used the samples for CFs established between 1997 and 2010, except for 1998, because the balance of potentially confounding variables of these samples was acceptable. Because the locations where CFs are likely to be established may change between years, we verified whether the SMD before matching depends on the CF establishment year when focusing on the SMD of the CF establishment year used in the subsequent analysis (i.e. from 1997 to 2010, except for 1998). No significant trends were observed (Table S3; Mann–Kendall rank correlation: *P* > 0.05), except for one potentially confounding variable (Mann–Kendall rank correlation: *P* = 0.04), which was no longer significant after Bonferroni correction. From the TCC and forest cover of matched samples, the longitudinal data set of TCC and forest cover from 8 years before CF establishment to 9 years after CF establishment was established. The longitudinal data set showed that both the TCC and forest cover of the CF and non-CF group consistently decreased, regardless of whether they were CFs, from 8 years before CF establishment to 9 years after CF establishment (Fig. 1; Table S4; Mann–Kendall rank correlation: *P* < 0.05). Even if we only examined the period after CF establishment, the declining trend remained the same (Table S5; Mann–Kendall rank correlation: *P* < 0.05). However, the ITSs applied to the differences of TCC and forest cover between CFs and non-CFs showed that the trajectories of TCC and forest cover in CFs after their establishment were different from those in non-CFs (Table S6). CF establishment was followed by a significant increase in TCC of 2.31 (*P* < 0.05) compared with non-CFs, i.e. the TCC of CFs is 2.31% higher than that of non-CFs at the time of CF establishment. The trend after CF establishment also showed that the difference in TCC between CFs and non-CFs significantly increased by 0.46% (*P* < 0.05) each year after CF establishment. There was no significant difference (*P* > 0.05) in forest cover after CF establishment, although the trend after CF establishment showed that the difference in forest cover between CFs and non-CFs significantly increased by 0.26% (*P* < 0.05).

To evaluate the longitudinal effect of CFs on forest degradation, we conducted statistical matching using the samples that were forest throughout the study period (between 1989 and 2019). Then, the longitudinal changes of TCC of the matched samples were analyzed. The longitudinal data set showed a decreasing trend in TCC for non-CFs after CF establishment (Table S5; Mann–Kendall rank correlation: *P* < 0.05), but no significant change for CFs after their establishment (Table S5; Mann–Kendall rank correlation: *P* > 0.05). The ITSs applied to the difference in TCC between CFs and non-CFs revealed that CF establishment was followed by a significant increase in TCC of 2.28% (*P* < 0.05) compared with non-CFs. The trend after CF establishment also showed that the difference in TCC between CFs and non-CFs significantly increased by 0.41% (*P* < 0.05).

We then assessed the effects of CFs on TCC and forest cover change after CF establishment for each initial land condition as of the CF's establishment year (Tables S7 and S8). We split the data set of TCC and forest cover depending on whether the samples were forest or nonforest as of the CF establishment year. When we used the data set of samples that were forest in the CF establishment year, CF establishment was followed by significant increases of 0.58 (*P* < 0.05) and 0.26 (*P* < 0.05) compared with non-CF for TCC and forest cover, respectively, representing the forest conservation effects of CFs. When we used the data set of samples that were nonforest in the CF establishment year, CF establishment was not followed by significant increases (*P* > 0.05) for TCC and forest cover compared with non-CF, which represented the nonsignificant forest recovery effects on CFs. When we used the matched samples derived from samples that were forest throughout the study period, CF establishment was followed by significant increases (*P* < 0.05) in TCC.

**Fig. 2.**
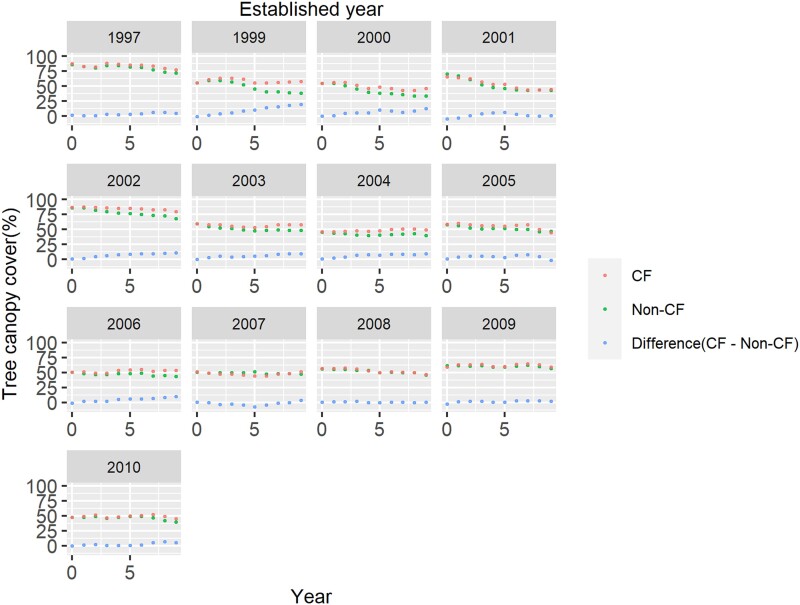
Temporal trajectories of TCC after CF establishment for samples that were forest in the year of CF establishment, aggregated by CF establishment year.

Finally, we assessed the effect of CF establishment year on deforestation and forest degradation and forest recovery (Tables 1 and 2). When we used the data set of samples that were forest in the CF establishment year, TCC and forest cover of CF and non-CF groups showed different trajectories depending on the CF establishment year; however, in general, CFs had higher forest cover and TCC (Fig. 2; Figs. S2 and S3). Regression analysis was conducted using the difference in TCC and forest cover between CFs and non-CFs as the objective variables, and the time since CF establishment, CF establishment year, and their interaction terms as explanatory variables. The regression results showed negative relationships between the interaction term and the objective variables both for TCC and forest cover (*P* < 0.05). When we used the data set of samples that were nonforest in the CF establishment year (Figs. S4 and S5), we found positive relationships in the interaction term (*P* < 0.05) for TCC, but the relationship for forest cover was not significant (*P* > 0.05). When the matched samples derived from only forest samples throughout the study period were used, the interaction term was not significantly linked to TCC (*P* > 0.05).

**Table 1.** Summary of regression analysis on the data set for the difference between CF and non-CF group by CF establishment year from matched samples that were forest as of CF establishment year.

	Tree canopy cover		Forest cover		Tree canopy cover of forest samples	
	Coefficients	Standard error	Coefficients	Standard error	Coefficients	Standard error
Intercept	125.585	283.632	−139.024	166.391	498.385	263.249
Year since CF establishment	166.305	53.129^a^	65.758	31.168^b^	72.701	49.311
Year CF was established	−0.062	0.142	0.069	0.083	−0.248	0.131
Year since CF establishment × year CF was established	−0.083	0.027^a^	−0.033	0.016^b^	−0.036	0.025

^a^ *P* < 0.01. ^b^*P* < 0.05.

**Table 2.** Summary of regression analysis on the data set for the difference between CF and non-CF group by CF establishment year from matched samples that were nonforest as of CF establishment year.

	Tree canopy cover		Forest cover	
	Coefficients	Standard error	Coefficients	Standard error
Intercept	2071.100	298.577^a,b^	161.127	96.351
Year since CF establishment	−172.549	55.929^b^	2.288	18.048
Year CF was established	−1.032	0.149^b^	−0.080	0.048
Year since CF establishment × year CF was established	0.086	0.028^b^	−0.001	0.009

^a^ *P* < 0.05. ^b^*P* < 0.01.

## Discussion

### Forest conservation effectiveness of CF

Previous studies have shown CFs result in forest conservation by preventing deforestation (18, 33, 34). However, the forest conservation effectiveness of CFs in terms of preventing forest degradation and facilitating forest recovery is still unknown. Our study provides the first country-level assessment of the effectiveness of CFs in preventing forest degradation and stimulating forest recovery. In addition, most previous studies that have examined the forest conservation effectiveness of CFs, especially in reducing deforestation and forest loss, have focused only on forest change since 2000 because of the limitations of their data sets (18, 20, 33). In contrast, the findings of the present study are based on long-term data from 1989 to 2019. Our results clearly suggest that CFs can prevent both forest degradation and deforestation because CF establishment mitigates the decrease of TCC and forest cover. CFs are often expected to contribute to global initiatives on reducing both deforestation and forest degradation, such as Reducing Emissions from Deforestation and Forest Degradation (REDD+) at the local level (35–37), while the evidence of contribution of CFs to reducing forest degradation is limited. Our study supports that CFs could be an effective option for implementing the global initiatives on reducing not only deforestation but also forest degradation.

Our analyses revealed that there was no significant difference in the nonforest matched samples in CFs and non-CFs in terms of both forest cover and TCC after CF establishment. The results indicate that effect of CFs on forest recovery cannot be verified, in contrast to the clear evidence of the effect of CFs in preventing forest degradation and deforestation. Furthermore, mean TCC and forest cover consistently decreased after CF establishment when the matched data set derived from whole samples was used. The results indicate that the outcome for forest recovery in CFs does not exceed deforestation and forest degradation. Forest restoration activities are often conducted in CFs in Cambodia after CF establishment, especially in their initial stages (38), but the efforts for forest restoration may have not reached a statistically detectable forest recovery. However, it is worth noting that the TCC of the samples that were forest throughout the study period showed a positive correlation after CF establishment, while it was not significant at the 5% level. The results imply that quality of forest condition in CFs may have improved, but that the effect had not yet reached detectable levels. Furthermore, because of data limitations, only forest recovery from the CF establishment year to 9 years after CF establishment was examined. An evaluation using longer-term data is also needed.

### Influence of CF establishment year on forest conservation

We also found that the forest conservation effect of CFs varied depending on CF establishment year. Further, recent CFs had little effect on preventing decreases in TCC and forest cover compared with older CFs. Similar results were found in Nepal (20). The study in Nepal tested whether there were differences in deforestation in CFs with different time periods since establishment using deforestation data from 2000 to 2012. The study found that older CFs were more effective for preventing deforestation. In our study, we assessed the effect of CF establishment year on deforestation and forest degradation from the CF establishment year to 9 years after CF establishment. Thus, unlike the study in Nepal, our study does not reflect the effect of time since establishment but reflects the effect of the establishment year itself. Various factors influence forest conservation performances of CFs (34, 39, 40); however, the causes of these differences in forest conservation performance by CF establishment year were beyond the scope of our study. One possibility is the lack of support. Because the number of CFs established was small at the beginning, there may have been enough support for each CF from NGOs, government agencies, and other organizations. Conversely, over time, the number of CFs increased and the available support for new CFs may have been insufficient. The decline of forest conservation effectiveness of CFs warns that the ineffective CFs in forest conservation may be increasing in Cambodia. In 2010, the Cambodian government announced a planned increase in the total area of CFs to 2 million ha by 2029 as part of the national forest program (31). Because the national forest program began in 2010, but our study only focused on CFs established before 2010 because of data availability, almost all CFs we evaluated in this study were established before the national forest program commenced. Therefore, our results do not necessarily reflect the forest conservation effectiveness of CFs established under the national forest program; therefore, assessment of these CFs is urgently required. Although our study is limited to Cambodia, the issues we have raised are applicable to other countries as well. This is because many CFs have been established not only in Cambodia but also in other tropical countries, including those elsewhere in Southeast Asia. In particular, Southeast Asian countries are increasing their CFs by setting target areas, as is the case in Cambodia (41). However, CFs are a means to forest management, not an end. Appropriate monitoring is needed to verify whether CFs with low conservation effectiveness are not increasing to ensure that the intended target areas are being met.

Studies examining the forest conservation effects of zoning approaches such as protected areas and CFs have increased since the 2000s [for example 42–45). Most studies have compared deforestation rates that have occurred over a period of time inside and outside given zoning, after accounting for the effect of potentially confounding variables. These studies have provided much insight into the effectiveness of zoning approaches. Conversely, existing methods make it difficult to compare forest conservation effectiveness before and after establishment and to evaluate differences in conservation effects depending on the year of establishment. Our approach can solve these problems in addition to evaluating the forest conservation effectiveness in terms of forest degradation and forest recovery.

A limitation of this study is that it did not analyze the relationship with other land cover zoning. Although this study compared the forest conservation effectiveness of CFs and non-CFs, this study excluded other major zoning areas, such as protected areas, from the analysis. In other words, this study compares the CFs with lands that are not assigned major zoning, and the relevance of the CFs to other zoning policies is unclear. For example, it is possible that the establishment of CFs may reduce the probability of being assigned to other land zoning. In Cambodia, deforestation resulting from large-scale land grabbing via zoning, called economic land concession (ELC), is a very serious problem, and it is possible that the allocation of CFs may result in some cases of being out of the zone of ELC. Taking these effects into account, it is possible that the recently established CFs may also have a forest conservation effect.

## Conclusion

CFs are used as a tool to achieve forest conservation; our results show that CFs can be effective as such, especially for preventing deforestation and forest degradation. However, contributions of CFs to reducing deforestation and forest degradation were linked to their establishment year, and recently established CFs contribute less to forest conservation than older ones. The results of this study are both hopeful and alarming regarding the potential of CFs and, ultimately, zoning policy in terms of positive conservation outcomes. Even if zoning areas such as CFs are increased because the existing zoning policy has been effective in forest conservation, it does not necessarily mean that the new zoning areas will be as effective as the existing ones. The effectiveness of the established zones should therefore be properly evaluated.

## Study area

Our study area covers the whole of Cambodia, a country with a total land area of approximately 18.1 million ha, located in Southeast Asia (Fig. S1). The forests of Cambodia have been facing relentless deforestation and forest degradation for decades (46, 47) but are vital for local livelihoods. Therefore, CFs were started in the 1990s as a strategy to achieve both forest conservation and local livelihood improvement. In this study, we used the CF data set obtained from the Forestry Administration, which is a part of the Ministry of Agriculture, Forestry and Fisheries of Cambodia. The data set includes the boundary and establishment year of each CF established between 1994 and 2010. It should be noted that there were no CFs established in 1996. Because the data sets were missing information for some CFs in the year when they were established, we excluded from analysis the CF areas for which the establishment year was missing. In addition, some CFs overlapped with water areas, protected areas, and ELC, and we therefore excluded them from the analysis (see next paragraph). Finally, we used the 426 CFs that were established between 1994 and 2010, of which the total area was 259,756.7 ha.

We excluded water areas, protected areas, and ELCs from the analysis. Previous studies around the world have shown that protected areas, on average, reduced deforestation (43, 44, 48, 49), while the effectiveness of protected areas varies as a result of a number of factors (45, 50). In Cambodia, protected areas reduced deforestation as in other countries (17), and thus, including protected areas in analyses may bias the results. ELCs are long-term land leases for economic development. Because the aim of ELCs are primarily agricultural intensification(51), the areas of ELC justifiably tend to convert forest to commercial agricultural land and show higher deforestation rates than the areas outside ELCs (52, 53). The effects of ELCs are beyond the scope of our study, but their inclusion would likely have biased our results. The boundaries for water areas, protected areas, and ELCs were downloaded from Open Development Cambodia (https://opendevelopmentcambodia.net/). We used “water bodies,” “natural protected areas in Cambodia,” and “economic land concessions” as the data sets for water areas, protected areas, and ELCs, respectively.

## Methods

### Data

We used the 30-m resolution TCC and forest cover change data set published by Shimizu et al. (54), which included annual TCC and forest cover change between 1989 and 2019 for the whole of Cambodia. TCC represents the tree canopy cover in a pixel (0–100%) in each year. Forest cover change data provide forest disturbance types and land cover classification in each year. Forest disturbance includes disturbance with land cover conversion of natural forest (forest to agriculture, forest to grass/shrub, forest to urban/water, and forest to plantation) and the disturbance without land cover conversion of natural forest. Land cover includes five classes, which were natural forest, plantation forest, agricultural land, grass/shrub, and urban/water. In this study, we reclassified the annual forest cover change data to annual forest cover data that represent forest/nonforest in each year. Natural forest and plantation forest were defined as forest, and the other classes including all disturbance classes were defined as nonforest.

We obtained the coordinates of villages and district centers from the National Institute of Statistics of the Ministry of Planning of Cambodia; the coordinates are based on the 2008 census. We obtained the line data set of main roads from the Ministry of Public Work and Transport of Cambodia. The elevation and slope were derived from a digital elevation model (DEM) of the Shuttle Radar Topographic Mission (55) obtained from the USGS archives (http://earthexplorer.usgs.gov/).

### Outcomes

TCC and forest cover were used as outcomes. The TCC estimated by Shimizu et al. (54) was used as the indicator of forest degradation and tree canopy recovery. Forest cover was the binary variable that represents forest and nonforest. We used annual forest/nonforest maps created from Shimizu et al. (54) and assigned 1 for forest and 0 for nonforest. The annual forest cover data for the analysis were used as the indicator of deforestation and forest recovery.

### Units of analysis

The spatial unit of this study was a grid cell with a 30-m resolution, which corresponded to the resolution of the forest cover change and tree cover density data set. There were approximately 130 million pixels inside the study area (i.e. the whole of Cambodia except for water areas, protected areas, and ELCs). We randomly selected 1% of the total samples for our analyses to avoid pseudoreplication effects.

Because we calculated forest areas in a 5-ha buffer in each sample for the potentially confounding variables (see the Potentially confounding variables section), we excluded the samples with buffers that were crossed by the border of Cambodia. In other words, we only selected the samples with buffers that were completely within Cambodia. The number of corresponding samples was 2,504. Finally, 1,113,636 samples, which comprised approximately 0.9% of the study area, remained. It should be noted that the samples with buffers that were crossed by water areas, protected areas, and ELCs were included in the analysis. We included forests in those areas when we calculated forest areas in a 5-ha buffer.

### Potentially confounding variables

Potentially confounding variables included elevation, slope, distance to the nearest main road, distance to the nearest village, distance to district centers, forest cover in CF establishment year, TCC in CF establishment year, distance from nonforest area in CF establishment year, and proportion of forest area around the samples in CF establishment year. Forest cover in CF establishment year was dependent on whether the sample was forest or nonforest in the CF establishment year. Distance from nonforest area was defined as the distance between the sample and the nonforest pixel, which was calculated using the “gridDistance” function in the *raste*r package of R (56). The “gridDistance” function calculates the nearest distance through a path connected from the center of a pixel to the centers of eight neighboring pixels. If the sample was nonforest in the CF establishment year, 0 was assigned for the distance from nonforest area in the CF establishment year. The proportion of forest area was defined as the proportion of forest area in the CF establishment year to the total land area within a 5-ha buffer (a radius of 126 m).

Elevation and slope were used as proxies for agricultural suitability and forest accessibility. Distance to the nearest main road, distance to the nearest village, and distance to district centers were used as the proxies for forest accessibility and for accessibility to markets. Sample forest cover, tree density, distance from nonforest area, and proportion of forest area were included to control for the initial forest conditions in CF establishment year.

### Matching

CF allocation is biased, because it is not necessarily geographically random, and this may influence the likelihood of outcomes. We thus applied matching techniques to compare TCC and forest cover changes between CFs and non-CFs to control for potentially confounding variables that affect both the location of CFs and outcomes. We defined the treatment group as samples inside CF boundaries and the control group as samples outside CF boundaries. Then, the randomly selected samples that were inside CF boundaries were matched with the samples that were outside CF boundaries.

Our data set included CFs with different establishment years between 1994 and 2010, and we used the forest conditions in the CF establishment year (i.e. forest cover, TCC, distance from nonforest area, and proportion of forest area around the samples in the CF establishment year) as potentially confounding variables. Thus, we used forest condition in different years for the matching techniques, depending on the CF establishment year. To accomplish this, we applied matching by CF establishment year. Specifically, matching was applied in phases from the oldest CFs to the youngest CFs. At first, we conducted matching using all samples that were inside CFs established in 1994 (i.e. samples located within CFs established in 1994) and all samples that were outside CFs established between 1994 and 2010 (i.e. all control samples). Forest conditions in 1994 and the other variables were used as the potentially confounding variables. Then, we conducted matching using the treatment samples of the second oldest CFs, and all samples outside the boundaries of CFs established between 1994 and 2010 that had not been matched in the initial matching. We repeated this procedure from the oldest CFs to the newest CFs. We used exact matching for forest cover of the samples and propensity score matching with the nearest neighbor for the other potentially confounding variables without replacement.

We assessed the balance of potentially confounding variables between treatment and control groups before and after matching using the SMD. The SMD was calculated as the mean difference between treatment and control potentially confounding variables standardized by the SD of treatment group (57, 58). We assumed that an absolute SMD after matching of less than 0.25 was acceptable, based on Stuart's study (58). Because we conducted matching in phases from the oldest CFs to the newest CFs, the SMD was calculated for each data set depending on CF establishment year. Matching decreased the SMD in each CF establishment year (Table S1). However, absolute SMDs in 1994, 1995, and 1997 were still larger than 0.25 after matching. We thus removed the matched samples where the absolute SMD was larger than 0.25. The final sample set included CFs established between 1997 and 2010, except 1998. The absolute SMD after matching was no greater than 0.02 when the sample from 1997 to 2010 (except 1998) was pooled, which provided sufficient bias correction (Table S1).

We extracted TCC and forest cover of matched samples in each CF establishment year from 1989 to 2019. Then, we converted TCC and forest cover from 1989 to 2019 to TCC and forest cover since CF establishment by subtracting CF establishment year from each year. In the control samples, the CF establishment year of matched treatment samples was used. Finally, a longitudinal data set of TCC and forest cover since CF establishment was created.

It is worth noting that the time range based on CF establishment year is dependent on the CF establishment year of the samples. For example, the time range was between 8 years before CF establishment and 22 years after CF establishment for CFs established in 1997, but the time range was between 21 years before CF establishment and 9 years after CF establishment for CFs established in 2010. Thus, when we analyze the pooled samples, we used the data from 8 years before CF establishment and 9 years after CF establishment, which was calculated from the entire matched samples.

Similar to the matching process described above, statistical matching was performed using only samples in forests that had been continuously maintained during the study period (from 1989 to 2019). Potentially confounding variables included elevation, slope, distance to the nearest main road, distance to the nearest village, distance to district centers, tree density in CF establishment year, distance from nonforest area in CF establishment year, and proportion of forest area around the samples in CF establishment year but did not include forest cover in CF establishment year. This is because the samples in the CF establishment year were assumed to be forest. Where an absolute SMD of less than 0.25 was allowed, the samples were included for a total of 11 years, in 1997 and from 2000 to 2010 (Table S2). This was because absolute SMDs of CFs in 1994, 1995, and 1997 were still larger than 0.25 after matching. The absolute SMD after matching was no greater than 0.03 when the sample from 1997 to 2010 (except 1998) was pooled, which provided sufficient bias correction (Table S2).

TCC from 1989 to 2019 of matched samples in each CF establishment year was extracted and converted to TCC from the year of CF establishment as in the method described above.

Analyses were conducted in R version 4.0.3 (59), and we used the *MatchIt* package (57) for propensity score matching.

### ITS analysis

ITS analysis, which is also a kind of event study, is a quasi-experimental method that evaluates the effect of an intervention from a time series outcome before and after the introduction of the intervention (32, 60). ITS analysis using only treatment data (i.e. CF groups in this case) is often employed; however, in this study, we aimed for a more robust analysis, so we included control data (i.e. non-CF groups). We followed the methodology of previous studies using ITS analysis that included treatment and control groups [e.g. 61, 62). For each outcome, we calculated the means of the treatment and control group (i.e. CF and non-CF groups) in each year. Then, a new series of dependent variables representing the difference between treatment and control group was generated from the means. Because our analysis evaluated two outcomes, which were TCC and forest cover, and because there are two different matchings for TCC, the dependent variables include the following:

1. Differences between CF and non-CF in means of yearly TCC.
2. Differences between CF and non-CF in means of yearly forest cover.
3. Differences between CF and non-CF in means of yearly TCC derived only from forest samples throughout the study period.

All the differences were calculated by subtracting mean values of non-CF from mean values of CF. The model for the ITS analyses can be expressed as follows:


$$\begin{array}{rl} O\;= & \;\beta_{0}+\beta_{1}\;\times \mathrm{time}+\beta_{2}\;\times \mathrm{CF}\;\mathrm{establishment}+\beta_{3}\\ & \times \mathrm{time}\;\mathrm{since}\;\mathrm{CF}\;\mathrm{establishment}+e, \end{array}\;$$ (1)

where *O* was outcome, and “time” was the time since the study started. For example, when the pooled samples were analyzed (8 years before CF establishment and 9 years after CF establishment), “time” was from −8 to 9. “CF establishment” was the binary variable representing the time period in which the CF was established (0 represents the time before CF establishment; 1 represents the time after CF establishment), “time since CF establishment” represents time elapsed since the CF was established, $\beta_{0}$, $\;\beta_{1}$, $\beta_{2}$, and $\beta_{3}$ were coefficients, and *e* was the error. In this study, we hypothesized that forest conservation recovery efforts started after CF establishment and the results of the efforts can be measurable from a year later of CF establishment. Thus, we assigned 0 to both “CF establishment” and “time since CF establishment” terms, when either was a CF establishment year.

### Regression analysis

The effects of CFs on TCC and forest cover change after CF establishment for each initial land condition as of the CF's establishment year was assessed using regression analyses. Five data sets were created for the regression analysis by dividing matched samples depending on whether or not the sample was forest as of the CF establishment year:

- TCC change after CF establishment for samples that were forest as of CF establishment year.
- Forest cover change after CF establishment for samples that were forest as of CF establishment year.
- TCC change after CF establishment for samples that were nonforest as of CF establishment year.
- Forest cover change after CF establishment for samples that were nonforest as of CF establishment year.
- TCC change after CF establishment for samples derived from only forest samples throughout the study period.

For the evaluation, we only used TCC and forest cover between the CF establishment year and 9 years after CF establishment. Then, as with the ITS analysis, the yearly means of TCC and forest cover of treatment and control groups were calculated. The difference between treatment and control group in each year was also generated from the yearly means. The differences were regressed against “time since CF establishment.”

Finally, we assessed the effect of CF establishment year on forest degradation, deforestation, and forest recovery. Yearly means of TCC and forest cover of CF and non-CF groups were calculated by CF establishment year. Then, the difference between CF and non-CF groups by CF establishment year was generated from the yearly mean values. For the evaluation, we only used TCC and forest cover between CF establishment year and 9 years after CF establishment, as in the ITS analysis. The differences were regressed against “time since CF establishment,” “CF establishment year,” and their interaction term. If CF establishment year affected forest degradation, deforestation, and forest recovery, the coefficient of time since CF establishment would vary depending on CF establishment year. Thus, we hypothesized that the interaction term between “time since CF establishment” and “CF establishment year” is the indicator of the importance of CF establishment year.

## Supplementary Material

pgad320_Supplementary_Data

## Data Availability

Tree canopy cover and forest cover change data were provided by Shimizu et al. (https://doi.org/10.1080/17538947.2022.2061618). The coordinates of villages and district centers were provided by the National Institute of Statistics of the Ministry of Planning of Cambodia. The line data set of main roads was provided by the Ministry of Public Works and Transport of Cambodia. CF data sets were provided by the Forestry Administration, which is a part of the Ministry of Agriculture, Forestry and Fisheries of Cambodia. A digital elevation model of the Shuttle Radar Topography Mission is freely available from http://earthexplorer.usgs.gov/. The boundaries for water areas, protected areas, and ELCs were downloaded from Open Development Cambodia (https://opendevelopmentcambodia.net/).
