# Supplementary material for: Forest conservation effectiveness of community forests may decline in the future: Evidence from Cambodia
Source: PNAS Nexus. 2023 Sep 28;2(10):pgad320. doi: 10.1093/pnasnexus/pgad320 (PMC10614049; doi:10.1093/pnasnexus/pgad320)
Supplement: pgad320_Supplementary_Data [file pgad320_supplementary_data.pdf]

Supplementary Information for

Forest conservation effectiveness of community forests may decline in the future

Miwa Ota, Tetsuji Ota, Katsuto Shimizu, Nariaki Onda, Vuthy Ma, Heng Sokh and Nobuya Mizoue

Tetsuji Ota
Email: ota.tetsuji.887@m.kyushu-u.ac.jp

This PDF file includes:

Figures S1 to S5

Tables S1 to S8

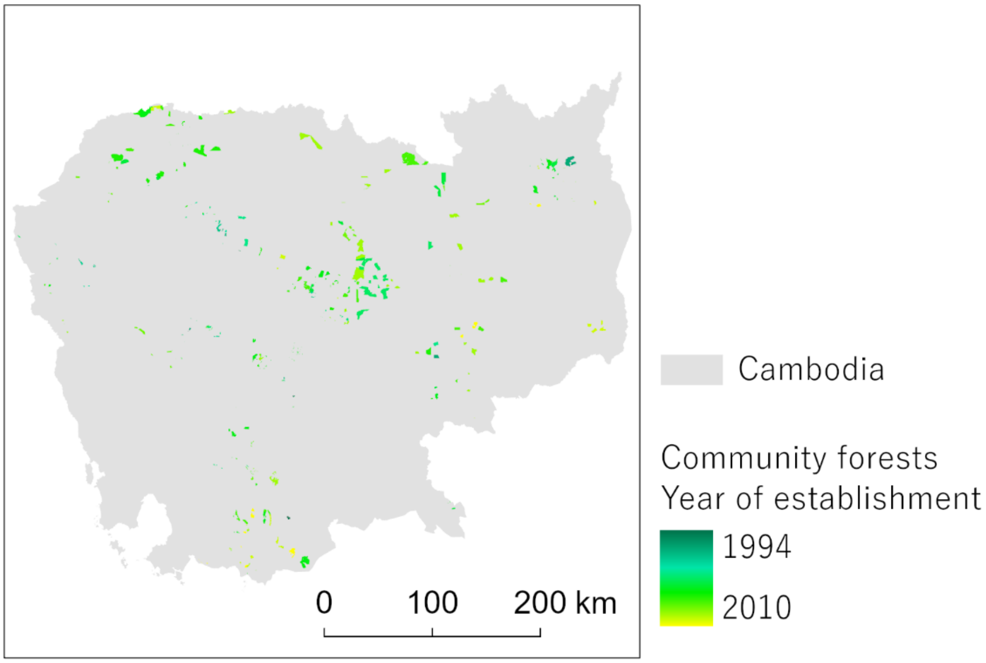

Fig. S1. Study area.

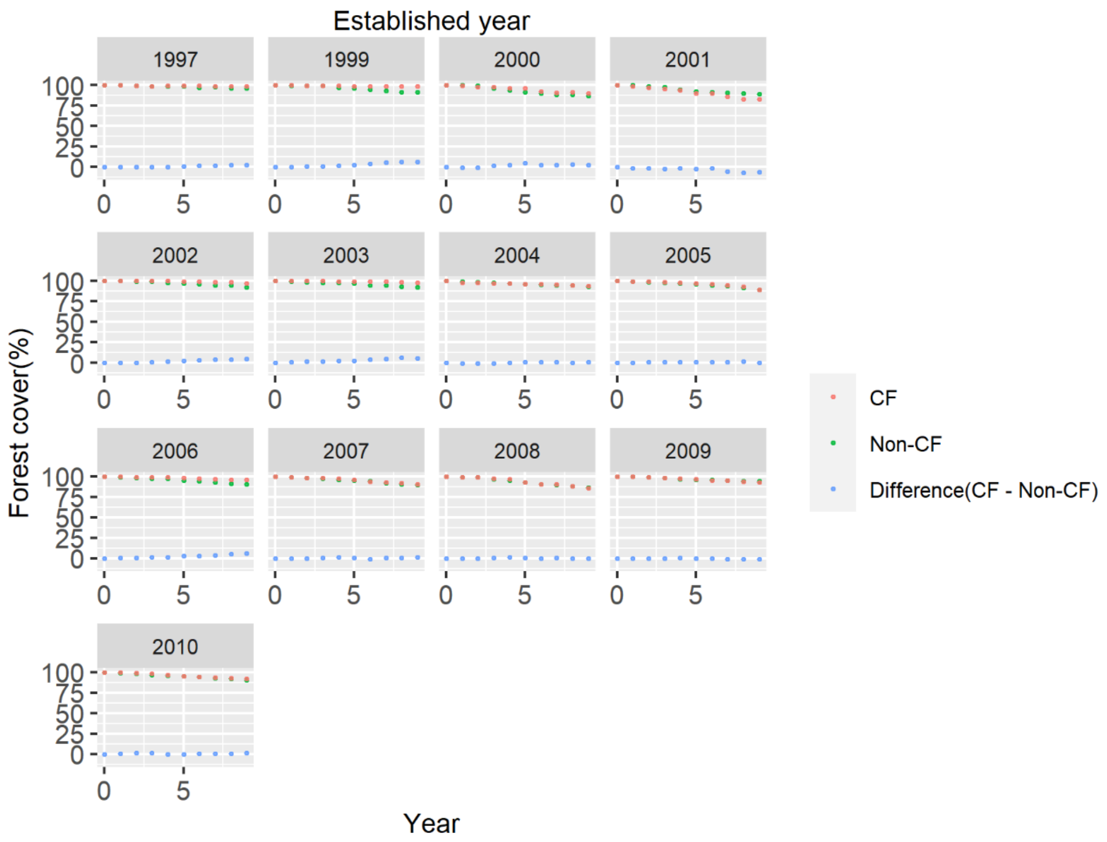

Fig. S2. Temporal trajectories of forest cover after community forest (CF) establishment for the matched samples that were forest in the year of CF establishment, aggregated by CF establishment year.

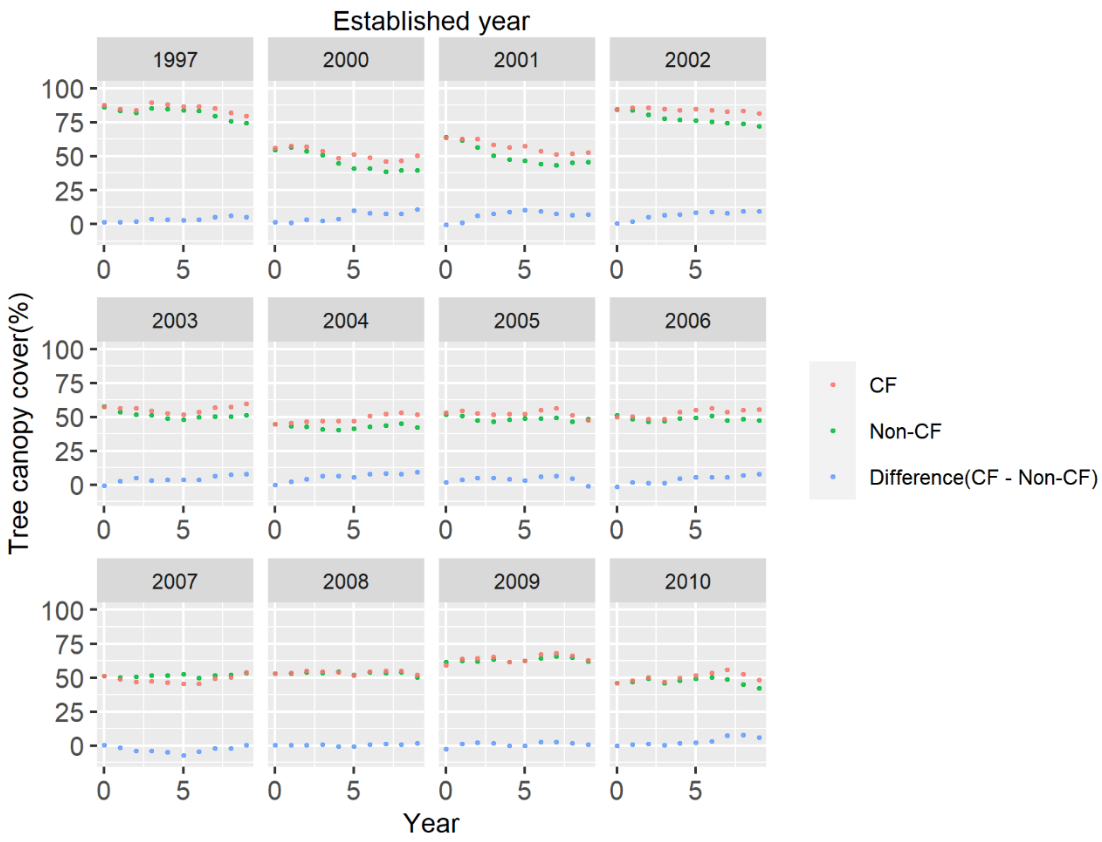

Fig. S3. Temporal trajectories of tree canopy cover after community forest (CF) establishment for the matched samples that were forest throughout the study period, aggregated by CF establishment year.

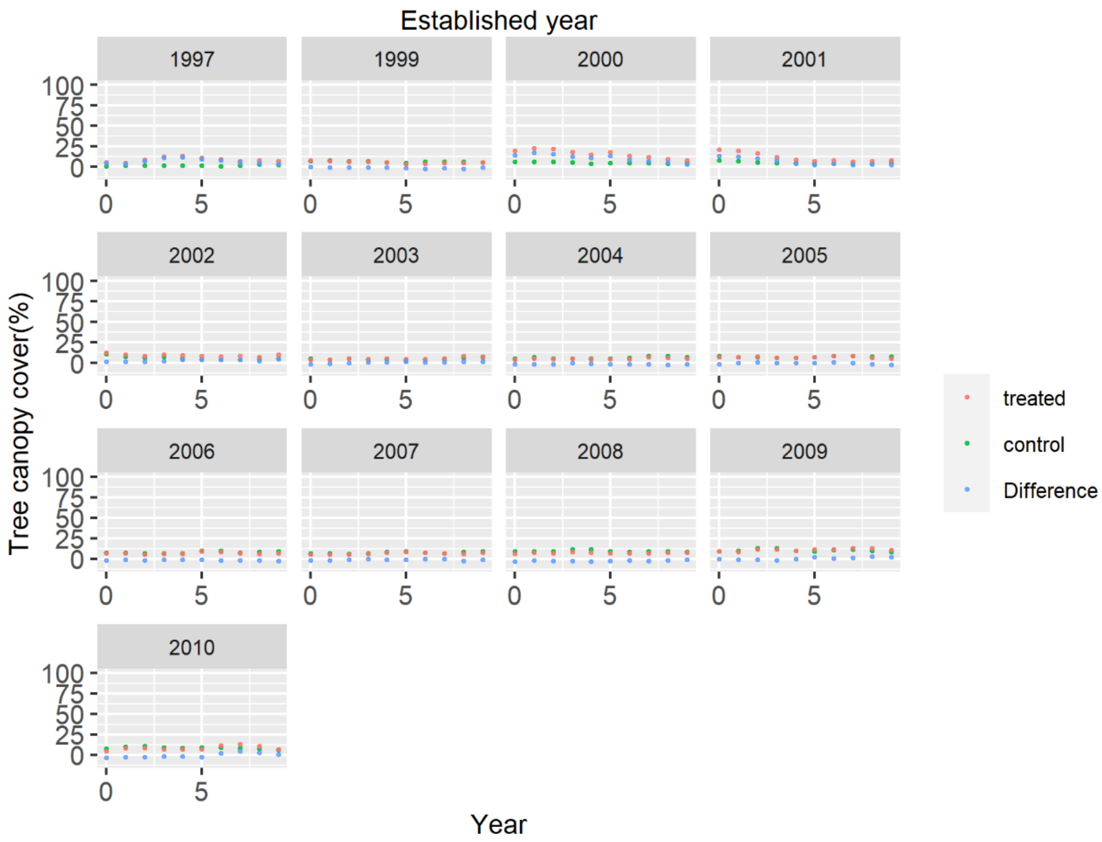

Fig. S4. Temporal trajectories of tree canopy cover after community forest (CF) establishment for the matched samples that were non-forest in the year of CF establishment, aggregated by CF establishment year.

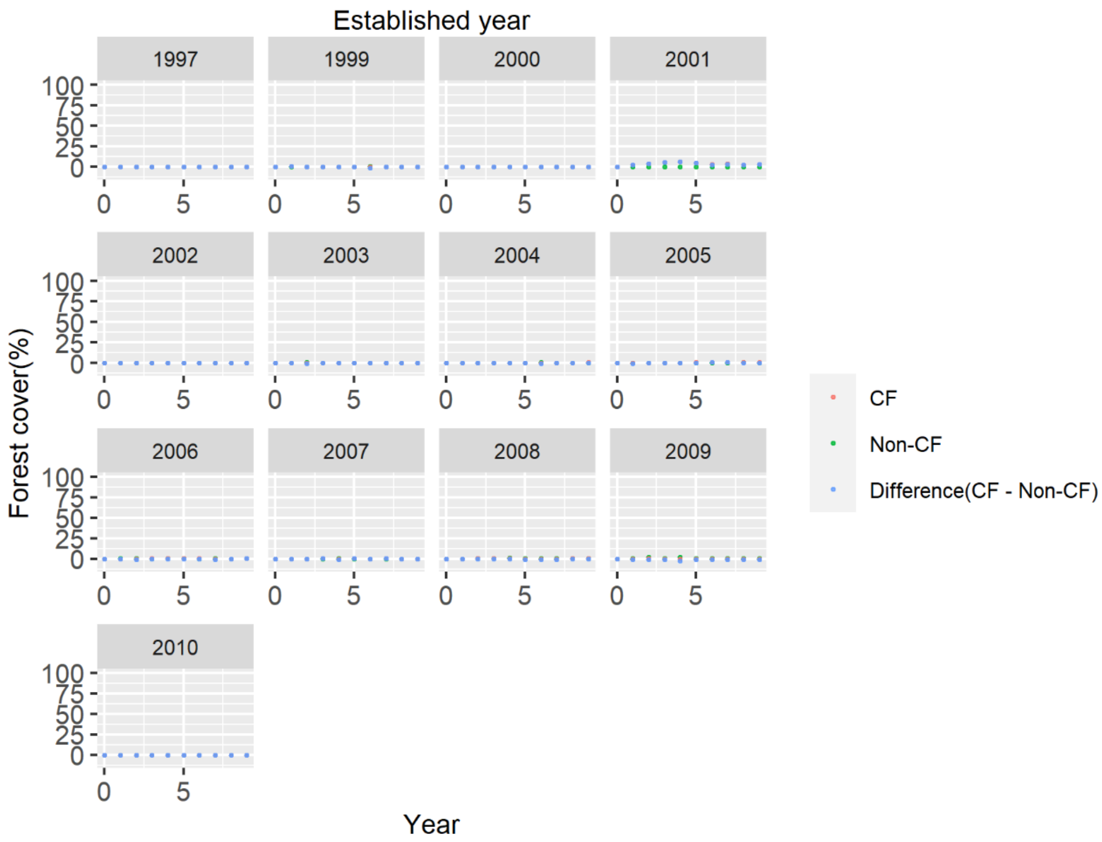

Fig. S5. Temporal trajectories of forest cover after community forest (CF) establishment for the matched samples that were non-forest in the year of CF establishment, aggregated by CF establishment year.

Table S1. Summary of standardized mean difference before and after propensity score matching.

CF established year	Before matching									After matching								
	Slope	Elevation	Road_DIST	Vill_DIST	DCent_DIST	ForNF_CFY	TC_CFY	NF_DIST	FArea_BUFF	Slope	Elevation	Road_DIST	Vill_DIST	DCent_DIST	ForNF_CFY	TC_CFY	NF_DIST	FArea_BUFF
1994	**-0.41**	**-10.49**	**-15.40**	**-7.36**	**-11.29**	**-1.78**	**-16.82**	**-40.89**	**-3.98**	0.14	**0.42**	**0.54**	**-0.63**	0.00	0.00	-0.03	0.00	-0.12
1995	**-1.06**	**-22.55**	**-18.47**	**-7.93**	**-9.48**	0.25	**-3.39**	**-4.60**	**0.30**	**-0.26**	**-2.18**	**0.48**	**-0.52**	**-0.82**	0.00	-0.20	**-0.63**	-0.19
1997	**0.87**	**1.29**	**-3.58**	-0.05	**-1.29**	**3.05**	**2.66**	**1.23**	**4.11**	-0.12	0.03	-0.08	0.03	0.12	0.00	0.07	0.00	0.05
1998	**-1.6**	**-1.37**	**-2.61**	**-1.47**	**-1.05**	**0.82**	**0.65**	**0.30**	**0.86**	0.04	**0.55**	-0.06	-0.03	**-0.41**	0.00	0.03	-0.06	0.09
1999	**0.32**	-0.16	**-0.90**	**-1.15**	-0.08	**0.84**	**0.57**	-0.05	**1.09**	0.10	0.13	0.04	0.07	0.09	0.00	0.00	0.00	0.04
2000	0.00	0.06	**-1.06**	**-1.18**	**-0.89**	**0.84**	**0.69**	-0.22	**1.09**	0.02	0.16	0.13	-0.09	0.13	0.00	0.11	-0.22	0.04
2001	-0.03	**-0.29**	**-0.80**	0.14	**-0.27**	**0.66**	**0.79**	0.04	**0.77**	0.05	0.02	-0.02	-0.04	0.01	0.00	0.01	0.01	0.00
2002	0.09	**0.84**	**2.53**	**0.45**	**1.25**	**3.96**	**2.55**	**1.09**	**5.96**	-0.02	-0.01	0.00	0.13	-0.01	0.00	0.04	0.01	0.07
2003	**0.33**	**0.40**	-0.21	**0.40**	0.24	**1.48**	**0.82**	**0.54**	**1.77**	-0.03	0.02	0.03	0.03	0.02	0.00	0.00	0.03	0.02
2004	**0.49**	**0.59**	**-0.59**	**0.34**	**-0.42**	**0.75**	**0.39**	0.08	**0.95**	0.06	0.04	-0.02	0.05	0.03	0.00	0.00	0.04	-0.05
2005	-0.03	**0.36**	0.04	**0.93**	**0.65**	**1.15**	**0.84**	**0.36**	**1.45**	0.01	0.04	0.00	0.00	0.01	0.00	0.02	0.03	0.01
2006	**0.30**	**0.59**	**1.14**	**0.76**	**1.01**	**1.64**	**0.87**	**0.43**	**2.20**	-0.02	0.00	-0.03	-0.04	0.04	0.00	-0.02	-0.02	-0.01
2007	**0.55**	**0.58**	**0.37**	-0.05	**0.32**	**1.26**	**0.82**	**0.31**	**1.69**	0.09	0.07	0.03	0.10	0.04	0.00	0.02	0.06	-0.01
2008	**0.39**	**0.44**	**0.85**	**0.58**	**0.27**	**1.55**	**0.92**	**0.37**	**2.17**	0.02	-0.03	-0.12	-0.01	-0.05	0.00	0.00	-0.03	0.03
2009	**0.80**	**1.13**	0.00	**0.54**	0.05	**2.17**	**1.33**	**0.58**	**3.08**	-0.07	0.02	0.01	-0.03	-0.02	0.00	-0.08	-0.17	-0.01
2010	**0.82**	**0.72**	-0.25	-0.18	0.02	**1.30**	**0.73**	**0.36**	**1.69**	0.05	0.02	0.05	0.12	0.04	0.00	-0.01	0.06	0.00
all (1997,1999–2010)	**0.69**	**0.51**	**0.61**	**0.38**	**0.45**	**0.90**	**0.94**	**0.58**	**0.99**	0.01	0.01	-0.02	0.01	0.00	0.00	0.00	0.00	0.00

*1 Road_DIST: distance to the nearest main road, Vill_DIST: distance to the nearest village, DCent_DIST: distance to district centers, ForNF_CFY: forest cover in CF established year, TC_CFY: tree density in CF established year,
NF_DIST: distance from non-forest area in CF established year, FArea_BUFF: proportion of forest area around the samples in CF established year.

*2 standardized mean differences larger than 0.25 or less than -0.25 are shown in bold text.

Table S2. Summary of standardized mean difference before and after propensity score matching for the samples that were forest throughout the study period.

	Before matching								After matching							
CF established year	Slope	Elevation	Road_DIST	Vill_DIST	DCent_DIST	TC_CFY	NF_DIST	FArea_BUFF	Slope	Elevation	Road_DIST	Vill_DIST	DCent_DIST	TC_CFY	NF_DIST	FArea_BUFF
1994	**-2.29**	**-50.40**	**-84.28**	**-43.63**	**-47.83**	**-276.40**	**NA**[*3]	**-6.56**	**1.46**	**1.35**	**3.77**	**1.04**	**1.22**	**-0.64**	**NA**[*3]	0.00
1995	**-3.27**	**-47.16**	**-26.83**	**-11.68**	**-13.70**	**-6.53**	**-11.44**	**-0.52**	**-0.33**	**1.87**	-0.02	-0.16	**1.59**	**0.32**	**0.29**	**0.33**
1997	**0.75**	**0.95**	**-5.69**	**-1.06**	**-2.85**	**2.16**	**1.15**	**1.31**	-0.02	0.07	-0.02	-0.01	-0.07	0.09	-0.05	0.05
1998	**-3.16**	**-5.93**	**-4.56**	**-2.87**	**-3.14**	**0.36**	**-0.29**	0.19	0.19	**0.28**	0.07	-0.07	0.24	0.03	0.10	0.01
1999	0.09	**-1.45**	**-1.72**	**-2.71**	**-1.56**	0.14	**-0.75**	0.12	0.05	-0.02	-0.01	-0.14	**-0.27**	-0.05	-0.02	-0.11
2000	**-0.43**	**-1.23**	**-2.12**	**-3.64**	**-1.67**	0.09	**-1.43**	0.08	-0.08	-0.14	0.10	-0.01	-0.14	0.05	0.00	-0.16
2001	**-0.25**	**-1.54**	**-1.86**	**-0.86**	**-1.01**	**0.33**	**-0.46**	0.12	0.06	-0.06	-0.01	-0.01	0.02	-0.01	-0.11	-0.06
2002	**-0.49**	**-0.78**	**2.04**	**-0.42**	**0.77**	**1.47**	**0.81**	**1.74**	-0.01	-0.02	-0.03	0.00	-0.03	0.02	0.00	0.05
2003	0.16	-0.15	**-0.91**	**-0.87**	-0.17	0.24	**0.35**	**0.54**	-0.09	0.00	0.02	0.05	0.04	-0.02	0.05	0.00
2004	**0.42**	0.13	**-1.40**	**-0.39**	**-1.31**	-0.04	**-0.32**	0.07	0.04	0.05	0.06	0.03	0.02	0.01	0.02	0.02
2005	**-0.35**	**-0.88**	**-0.59**	**0.35**	-0.11	**0.27**	-0.08	**0.32**	0.01	0.05	0.04	-0.01	0.00	0.06	0.03	0.01
2006	0.14	-0.20	**0.87**	**0.26**	**0.74**	0.18	-0.17	**0.59**	-0.01	-0.06	-0.09	-0.13	0.00	-0.05	-0.09	-0.02
2007	**0.47**	0.06	0.07	**-1.00**	**-0.28**	**0.31**	**-0.31**	**0.51**	0.10	0.04	-0.01	0.06	0.03	0.01	0.02	-0.11
2008	**0.26**	-0.05	**0.52**	-0.08	-0.16	**0.36**	**-0.25**	**0.51**	-0.01	-0.07	-0.13	-0.09	-0.07	0.00	-0.07	0.05
2009	**0.74**	**1.02**	**-0.64**	-0.18	**-0.68**	**0.65**	**0.26**	**1.00**	-0.04	0.04	0.04	-0.01	0.00	-0.10	-0.15	-0.05
2010	**0.78**	0.09	**-0.80**	**-1.34**	**-0.47**	0.07	**-0.46**	**0.57**	-0.02	-0.06	-0.05	-0.03	-0.01	0.00	0.05	-0.02
all (1997,2000–2010)	**0.34**	0.04	0.25	-0.10	0.02	**0.34**	0.16	**0.30**	0.00	-0.01	-0.03	-0.03	-0.01	0.00	-0.01	0.00

*1 Road_DIST: distance to the nearest main road, Vill_DIST: distance to the nearest village, DCent_DIST: distance to district centers, ForNF_CFY: forest cover in CF established year,
TC_CFY: tree density in CF established year,
NF_DIST: distance from non-forest area in CF established year, FArea_BUFF: proportion of forest area around the samples in CF established year.

*2 standardized mean differences larger than 0.25 or less than -0.25 are shown in bold text.

*3 Since the standard deviation of treated group was 0, the SMD could not be calculated.

Table S3. Mann-Kendall rank correlation coefficient between year and standardized mean difference before matching from 1997 to 2010, excluding 1998.

Potentially confounding variables	Coefficients	
Slope	0.32	
Elevation	0.25	
Road_DIST	0.44	*
Vill_DIST	0.27	
DCent_DIST	0.26	
ForNF_CFY	0.17	
TC_CFY	0.14	
NF_DIST	0.14	
FArea_BUFF	0.16	

* $p < 0.05$

[1] Road_DIST: distance to the nearest main road, Vill_DIST: distance to the nearest village, DCent_DIST: distance to district centers, ForNF_CFY: forest cover in CF established year, TC_CFY: tree density in CF established year, NF_DIST: distance from non-forest area in CF established year, FArea_BUFF: proportion of forest area around the samples in CF established year.

Table S4. Mann-Kendall rank correlation coefficient between year and tree canopy cover and forest cover.

Variables	Groups	Coefficients	
Tree canopy cover	CF	-0.90	**
Tree canopy cover	Non-CF	-1.00	**
Forest cover	CF	-1.00	**
Forest cover	Non-CF	-1.00	**
TCC of forest samples	CF	-0.23	
TCC of forest samples	Non-CF	-0.92	**

* $p < 0.05$; ** $p < 0.01$

Table S5. Mann-Kendall rank correlation coefficient between year and tree canopy cover and forest cover after community forest (CF) establishment.

Variables	Groups	Coefficients	
Tree canopy cover	CF	-0.78	**
Tree canopy cover	Non-CF	-1.00	**
Forest cover	CF	-1.00	**
Forest cover	Non-CF	-1.00	**
TCC of forest samples	CF	0.38	
TCC of forest samples	Non-CF	-0.73	**

* $p < 0.05$; ** $p < 0.01$

Table S6. Results of interrupted time series analysis.

	Tree canopy cover			Forest cover			Tree canopy cover of forest samples		
	Coefficients	Standard error		Coefficients	Standard error		Coefficients	Standard error	
Intercept	-0.627	0.360		-0.001	0.078		-0.765	0.414	
Time	-0.044	0.076		-0.030	0.016		0.055	0.087	
CF establishment	2.311	0.557	**	-0.051	0.121		2.282	0.641	**
Time since CF establishment	0.457	0.107	**	0.264	0.023	**	0.405	0.123	**

* $p < 0.05$; ** $p < 0.01$

Table S7. Summary of regression analysis on the dataset for the difference between community forest (CF) and non-CF groups from matched samples that were forest as of CF establishment year.

	Tree canopy cover			Forest cover			Tree canopy cover of forest samples		
	Coefficients	Standard error		Coefficients	Standard error		Coefficients	Standard error	
Intercept	1.301	0.355	**	-0.046	0.092		0.951	0.330	*
Year since CF establishment	0.580	0.067	***	0.260	0.017	***	0.549	0.062	**

* $p < 0.05$; ** $p < 0.01$

Table S8. Summary of regression analysis on the dataset for the difference between community forest (CF) and non-CF groups from matched samples that were non-forest as of CF establishment year.

	Tree canopy cover		Forest cover	
	Coefficients	Standard error	Coefficients	Standard error
Intercept	-0.093	0.141	0.048	0.087
Year since CF establishment	-0.061	0.026	0.031	0.016

* $p < 0.05$; ** $p < 0.01$
